# Anti-Tumor Immunity and Preoperative Radiovaccination: Emerging New Concepts in the Treatment of Breast Cancer

**DOI:** 10.3390/ijms24119310

**Published:** 2023-05-26

**Authors:** Ioannis M. Koukourakis, Marios Papadimitriou, Dimitra Desse, Anna Zygogianni, Christos Papadimitriou

**Affiliations:** 1 Radiation Oncology Unit, 1st Department of Radiology, Aretaieion University Hospital, School of Medicine, National and Kapodistrian University of Athens, 11528 Athens, Greece; ikoukourakis@med.uoa.gr (I.M.K.); dimidesse@gmail.com (D.D.); azygogianni@med.uoa.gr (A.Z.); 2 Oncology Unit, Aretaieion University Hospital, School of Medicine, National and Kapodistrian University of Athens, 11528 Athens, Greece; marios_papadim@hotmail.com

**Keywords:** breast cancer, neoadjuvant chemotherapy, immunotherapy, radiotherapy, radiovaccination

## Abstract

Neoadjuvant chemotherapy (NACT) for certain breast cancer (BC) subtypes confers significant tumor regression rates and a survival benefit for patients with a complete pathologic response. Clinical and preclinical studies have demonstrated that immune-related factors are responsible for better treatment outcomes, and thus, neoadjuvant immunotherapy (IO) has emerged as a means to further improve patient survival rates. Innate immunological “coldness”, however, of specific BC subtypes, especially of the luminal ones, due to their immunosuppressive tumor microenvironment, hinders the efficacy of immune checkpoint inhibitors. Treatment policies aiming to reverse this immunological inertia are, therefore, needed. Moreover, radiotherapy (RT) has been proven to have a significant interplay with the immune system and promote anti-tumor immunity. This “radiovaccination” effect could be exploited in the neoadjuvant setting of BC and significantly enhance the effects of the already established clinical practice. Modern stereotactic irradiation techniques directed to the primary tumor and involved lymph nodes may prove important for the RT-NACT-IO combination. In this review, we provide an overview and critically discuss the biological rationale, clinical experience, and ongoing research underlying the interplay between neoadjuvant chemotherapy, anti-tumor immune response, and the emerging role of RT as a preoperative adjunct with immunological therapeutic implications in BC.

## 1. Introduction

Breast cancer (BC) is the second-most common malignancy affecting women in the US, with more than 250,000 cases reported annually [1]. Early BC patients are treated with primary surgery followed by adjuvant chemotherapy and/or hormonal therapy—when disease characteristics and molecular assays are indicative—and adjuvant radiotherapy (RT). In advanced carcinomas, such as inflammatory breast tumors, T4-staged cases, or cases with extensive nodal involvement, NACT is recruited with the goal of achieving tumor resectability, eradicating the potential micrometastatic spread, and eventually improving the overall survival (OS) of patients. Furthermore, in patients with operable BC candidates for adjuvant chemotherapy, NACT may be applied if breast conservation is difficult to achieve due to size of the tumor relative to that of the breast. NACT is also indicated for patients with operable disease but with unfavorable histology, e.g., HER2+ and triple-negative BC (TNBC), as NACT leads to higher rates of pathologic complete response (pCR), and eventually to favorable disease-free (DFS) and OS [2].

Unlike other tumors, RT does not have an established position in the neoadjuvant setting in BC. Nevertheless, developments, including the recent approval of the immune checkpoint inhibitor (ICI) pembrolizumab in combination with chemotherapy as a neoadjuvant therapy and its continued administration as a single agent adjuvantly after surgery for patients with high-risk, early-stage TNBC, together with the shifting focus toward unveiling and “tapping” into the radiovaccinating properties of irradiation, could provide the basis for reinventing neoadjuvant RT as a means to increase immunotherapy’s (IO) potency, aside from its direct and strong anti-tumor effectiveness.

In this review article, we provide an overview and critically discuss the biological rationale, clinical experience, and ongoing research underlying the interplay between NACT, the anti-tumor immune response, and the emerging role of RT as a preoperative adjunct with immunological therapeutic implications in BC.

A literature search was performed in the EMBASE and MEDLINE databases. The terms used to retrieve the reported studies were as follows: “breast cancer”, “neoadjuvant chemotherapy”, “radiotherapy”, “immunotherapy”, “preoperative radiotherapy”, “abscopal effects”, “immune response”, “immunogenic cell death”, and “tumor microenvironment”. Randomized phase III trials and selected phase II and translational studies were considered for the analysis.

## 2. Neoadjuvant Systemic Therapy for BC

Neoadjuvant systemic therapy for early-stage BC has evolved over many decades [3]. The National Surgical Adjuvant Breast and Bowel Project NSABP B-18 trial randomized 1523 patients to receive four cycles of doxorubicin/cyclophosphamide preoperatively or postoperatively [4]. Similar results, as far as DFS and OS are concerned, were reported for both treatment arms. In the NACT arm, 80% of tumors responded well to chemotherapy (26% with pCR) [5]. An interesting observation was that patients with pCR had a significantly better survival rate. The EORTC trial 10902, published in 2001, comprised 698 patients to receive NAC with FEC vs. the same regimen administered after surgery; it was demonstrated that, although 23% of patients exhibited a down-staging of their disease, the OS and progression-free survival did not improve with NACT [6]. Furthermore, the NSABP-B27 trial showed that the addition of taxanes in the preoperative regimen significantly increased the rate of pCR, and these patients had a better overall prognosis [7]. Similarly, the GEPARDUO study, in which a dose-dense chemotherapy regimen—including docetaxel—was applied, reported that patients with pCR had better long-term survival rates [8]. The European Cooperative Trial in Operable Breast Cancer published the results of a randomized trial in 2009 of 1355 women receiving paclitaxel-containing chemotherapy as an adjuvant, or NACT. Tumor response was noted in 78% of patients after NACT. Although relapse-free survival was not significantly improved compared to adjuvant chemotherapy, a two-fold higher achievement of breast-conserving surgery was possible due to NACT [9].

Further trials utilized pCR as a surrogate end-point for longer outcomes. Trastuzumab-based therapy for HER2+ BC was soon added in the neoadjuvant setting with increased efficacy, while dual blockade of the HER2 receptor with pertuzumab/trastuzumab combined with docetaxel was significantly associated with better pCR rates [2]. This benefit was also proven to be the case for locally advanced or inflammatory BC. As preoperative systemic treatment led to significant tumor reduction, breast conservation rates were also improved. However, it has to be underlined that pCR rates have been demonstrated to be higher in patients with HER2+ BC or TNBC [10], suggesting that factors beyond tumor pathology underlie this finding. HER2+ BC patients appear to benefit in terms of response due to the already discussed targeting agents. For TNBC, carboplatin and bevacizumab have been successfully applied in the neoadjuvant setting, providing pCRs of up to 53–67% [11,12]. A recent meta-analysis, however, has suggested that pCR is a far from ideal surrogate end-point for DFS and OS and should be replaced in the context of neoadjuvant trials for early BC [13].

## 3. Unveiling the Role of the Immune Response in pCR

The reason for the increased sensitivity of TNBC and HER2+ BC to chemotherapy is obscure. TNBC is a heterogeneous group of breast tumors. Subgroups linked to BRCA, FOXA1-related genes, or AR mutations seem to respond better to chemotherapy [14,15]. AKT1 overexpression, eventually linked to metabolic and autophagy pathways, as well as PD-1 enhanced expression, have been associated with poor prognosis in TNBC [16]. A feature, however, that discriminates TNBC and HER2+ BC from the luminal subtypes is their immunogenic potential. In an analysis of 3771 patients treated with NACT, a high tumor-infiltrating lymphocyte (TIL) density was noted in 13%, 19%, and 30% of luminal HER2−, HER2+, and TNBC, respectively [17]. pCR rates were directly related to a high TIL density (44% vs. 20–27%). This association was verified in all histological subtypes. In fact, luminal/HER2– cases bearing a high TIL density had a similar pCR to other tumor subtypes with a low/intermediate TIL density. Nevertheless, HER2+ BC and TNBC with a high TIL density exhibited a significantly higher pCR compared to “hot” luminal tumors. In a meta-analysis of 13 studies, high lymphocytic infiltration was linked with increased pCR rates only in HER2+ BC and TNBC [18].

It is, therefore, suggested that immunologically “hot” BCs have a better response to NACT, which could be attributed to immune-related factors. Such tumors also have a better prognosis [17]. Whether IO could improve pCRs and the survival of these patients has emerged as a hypothesis.

## 4. Toward ICIs in Combination with NACT

The use of ICIs targeting the PD-1/PD-L1 pathway appeared to be an interesting approach to further increasing pCRs. The KEYNOTE-522 trial comprised 1174 TNBC patients with stage II/III disease who received NACT with doxorubixine/cyclophosphamide with or without pembrolizumab (2:1 randomization) [19]. A significantly increased pCR rate (64.8% vs. 51.2%) in favor of the IO arm was noted. Stratification according to PD-L1 expression showed that pembrolizumab enhanced the pCR rates in patients with positive PD-L1 (68.9 vs. 54.9%) and with negative PD-L1 expression (45.3% vs. 30.3%). At the first planned analysis, the progression rates at any site with a median follow-up of 15.5 months were 7.4% in the pembrolizumab–chemotherapy vs. 11.8% in the placebo–hemotherapy arm, with distant recurrences being the most common event. A recent publication on another primary end-point of KEYNOTE 522, event-free survival (EFS), showed that the estimated EFS at 36 months was 84.5% in the pembrolizumab–chemotherapy group and 76.8% in the placebo–chemotherapy group, while the median event-free survival was not reached in either group [20].

Similarly, in an ongoing randomized phase II trial (I-SPY2), pembrolizumab combined with upfront chemotherapy was shown to achieve higher pCRs in stage II-III MammaPrint-high-risk luminal/HER2− BC and TNBC [21]. Specifically, the pCR rates in TNBC increased from 22% to 60% and in luminal/HER2− BC from 13% to 30% of cases. Complete responders displayed better event-free survival in all groups. The IMpassion 031 randomized trial included 333 patients with stage II/III TNBC to receive NACT with Nab-paclitaxel followed by doxorubicine/cyclophosphamide chemotherapy, with or without atezolizumab [22]. In accordance with the previous studies, the pCRs were improved in the IO arm (57.6% vs. 41.1%). The effect of IO on the survival of patients was, however, too early to assess.

Table 1 summarizes key randomized trials on neoadjuvant systemic therapy for BC patients.

## 5. Investigative Policies to Reverse Immunological “Coldness”

Beyond ICI inclusion in the NACT regimens, an additional important concept that emerged from the above discussed trials is the clinical value of a reversal of immunological “coldness”, eventually for all subtypes of BC. BC cells certainly express “foreign” antigens. However, 56%, 44%, and 29% of luminal/HER2−, HER2+ BCs, and TNBC, respectively, are deprived of any lymphocytic infiltration [17]. An investigation into the reasons for this discrepancy may provide biological insights with eventual therapeutic implications.

An overexpression of immune checkpoint inhibitory molecules, such as PD-L1, CTLA4, CD47, and other poorly investigated ones, prevents the cytotoxic activity of T cells and macrophages. The accumulation, however, of these immune cells, and their proliferation and activity depend on the complex state of the intratumoral microenvironment. Giatromanolaki et al. recently showed that about 25% of BCs have intense expression of LDH5 and HIF1α, which is suggestive of a hypoxic tumor microenvironment that, under the anaerobic metabolism conferred by LDHA gene upregulation, also becomes acidic [23]. This metabolic phenotype was significantly linked with poor TIL density, poor tertiary lymphoid structure formation, and an ominous prognosis. Inhibiting glycolysis, blockage of the HIF-pathway, reversal of intratumoral acidosis with LDHA, or carbonic anhydrase CA9 inhibitors may prove important for the reversal of the “coldness” of BCs in this subgroup of tumors [24,25,26].

Other microenvironmental conditions contributing to the development of immunologically inert tumors relate to the accumulation of immunosuppressive metabolites. Ectonucleotidase CD73 and CD39 overexpression by cancer and stroma cells metabolize that abundantly secreted by cancer cells ATP in the tumor stroma to adenosine, a potent suppressor of T-cell proliferation and activity of T cells, macrophages, and NK-cells [27]. In a recent study of lung cancer, an overexpression of CD39 was noted in 80% of tumors, which was linked with hypoxia, high PD-L1 expression, and the accumulation of regulatory FOXP3+ lymphocytes [28]. Clinical trials are ongoing to investigate the immunotherapeutic role of agents targeting adenosine receptors, CD73 or CD39, in combination with ICIs [26]. Kynurenine is another immunosuppressive metabolite produced from tryptophan under the enzymatic activity of indoleamine deoxygenase IDO1 [29]. The overexpression of IDO1 has been linked with luminal subtypes and poor prognosis in BC [30]. Inhibitors of IDO1 are under clinical investigation [29]. The depletion of arginine, a semi-essential aminoacid, from the tumor microenvironment, through the enzymatic activity of arginases, is also an important immunosuppressive pathway exploited by tumors [31]. Arginase inhibitors have been shown to suppress the metastatic potential of BC in experimental models [32].

The above policies to reverse immunological inertia may prove to be of importance in the enhancement of the activity of ICIs in the context of neoadjuvant treatment for BC.

## 6. Radiovaccination as a Means of Enhancing the Immunological Response

Cytotoxic cancer treatment modalities, including chemotherapy and RT, confer different types of cell death, with the most prominent ones being apoptosis and mitotic catastrophe [33,34]. Dying tumor cells have been shown to be responsible for the release of damage-associated molecular patterns (DAMPs) through which an anti-tumor adaptive immune response is elicited. This is the so-called immunogenic cell death (ICD) [35]. Chemotherapy and RT have been demonstrated to elicit ICD [36,37]. Thus, it is suggested that the direct effects of chemotherapy and RT are further prolonged by an immunity-sustained response toward malignant tumors.

Several studies have highlighted the significance of ICD in BC as a key factor that could be exploited for improved treatment results [38]. The mechanisms involved in ICD are complex and involve different triggering factors and biological pathways. In particular, the calreticulin calcium-binding protein residing in the lumen of the endoplasmic reticulum is translocated to the plasma membrane of damaged cells, providing an “eat me” signal to macrophages and dendritic cells (DCs) [39]. Moreover, calreticulin binds to DCs promoting tumor antigen presentation and cytotoxic T-cell activation [40]. Another important ICD-related event is the extracellular release of ATP in the preapoptotic stage following irradiation or exposure to chemotherapy [41]. ATP acts as a chemokine-like signal for DCs via binding to specific purinergic receptors [42]. Furthermore, the high mobility group box 1 (HMGB1) protein is passively released in the extracellular space during cancer cell apoptosis. HMGB1 binds to specific receptors such as RAGE and TLR4 on myeloid cells and facilitates the presentation of antigens by DCs [43]. In addition, cancer cell irradiation and chemotherapy induce the interferon-type-I response and expression of IFN-stimulated genes by damaged cells, promoting chemoattraction and proliferation of T-cells [44].

Ever since the rapid surge of cancer IO, RT’s immunostimulatory properties, revealed through a century of studies, have been revisited with renewed interest, and the focus has shifted toward the mechanisms through which RT can actually confer the so-called “radiovaccination” effect [45]. This involves dendritic cell priming, the enhanced recruitment of effector T cells, the modulation of immune checkpoint molecules, and HLA-class I expression. RT effects on the tumor microenvironment, e.g., cell adhesion molecule expression by endothelial cells that facilitate T-cell transmigration [46], further promote the immune system’s access to the tumor. Moreover, the release of chemokines by irradiated cancer cells can activate helper and cytotoxic T cells and also provide chemotactic stimuli to attract inflammatory cells [45]. The abscopal effects of RT, a term introduced by Mole RH in 1953 [47], can be explained through the above mechanisms. While clinical reports on non-irradiated metastases’ eradication after localized RT are numbered, one must not overlook the immunosuppressive properties of irradiation, mainly concerning the upregulation of immune checkpoint molecules such as PD-L1 on cancer cells that leads to T-cell neutralization. In addition, large-field RTs may significantly compromise the overall lymphocytic content in the bloodstream and the irradiated lymph nodes, which may prove detrimental for both RT and IO. A combination, however, of ICIs and limited-field stereotactic RTs could eventually unmask the vaccine features of RT and launch a new era of cancer treatment [48].

Several preclinical studies investigated this combined treatment for BC with positive results. One or two fractions of 12 Gy delivered to murine breast carcinoma together with an anti-CTLA4 antibody led to improved survival in mice when compared with RT or anti-CTLA4 therapy alone [49]. The same research group suggested that abscopal effects were evident in similarly treated TSA mouse mammary cancers when fractionated RT (three fractions of 8 Gy or five fractions of 6 Gy) was delivered instead of a single 20 Gy fraction [50]. This conclusion was later confirmed by Vanpouille-Box et al. [51] in a study that displayed poor activation of the IFN-type-I pathway with single fraction radiation doses above 18 Gy. In accordance with the already mentioned effects of RT on immune checkpoint molecules, the irradiation of BC on mice was responsible for PD-L1 upregulation on cancer cells, while treatment with an anti-PD-L1 antibody augmented the immune-mediated cytotoxic effects of RT [52].

There are not adequate clinical data on RT and IO for BC as of yet. Published small phase II trials have focused on metastatic TNBC or HER2+ carcinomas. Three fractions of 8 Gy followed by nivolumab were linked with a poor response in metastatic TNBC patients [53], and that was also the case for ER+ and HER2− patients that received RT (20 Gy/5 Gy/fraction) together with pembrolizumab [54]. On the other hand, concurrent whole-brain RT, anti-CTLA-4 tremelimumab, and trastuzumab conferred a 12-week non-central nervous system disease control rate of 33% with good tolerance [55]. In addition, Ho et al. demonstrated that concurrent RT (30 Gy/6 Gy/fraction) and pembrolizumab achieved three complete responses in 17 patients with metastatic TNBC [56].

**Figure 1:**
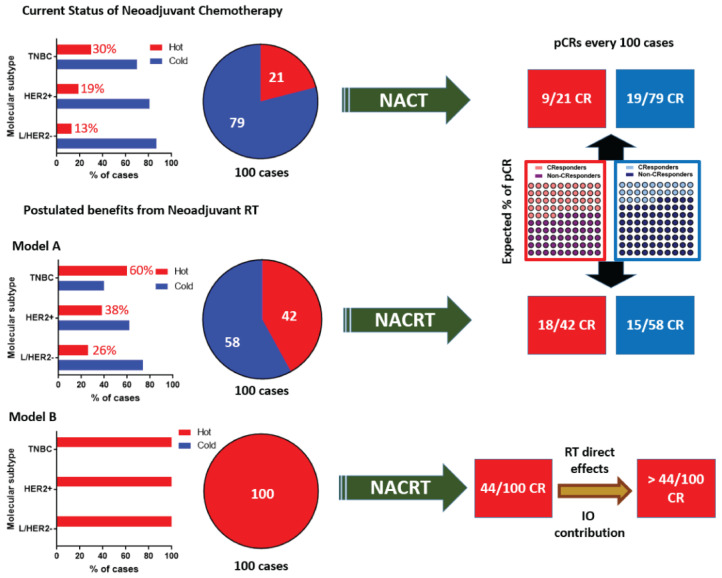
Schematic presentation of the speculated benefit of radiovaccination expected from the combination of neoadjuvant radiotherapy (RT) with neoadjuvant chemotherapy (NACT). The model is based on indicative percentages (with approximation) reported in the study by Denkert et al. [14], where high tumor-infiltrating lymphocyte (TIL) density was noted in 13%, 19%, and 30% of luminal HER2−-(L/HER2−), HER2+, and TNBC, respectively, which account for an average of 21 out of 100 BCs with high TIL density. The pathologic complete response (pCR) rates reported were 44% in “hot” (high TIL density) and 25% in “cold” (low-intermediate TIL density) BCs, regardless of the molecular subtype. We assumed a doubling of the immunologically “hot” tumors due to irradiation to provide the Model A (Model A; 42/100 BCs). Following this assumption, neoadjuvant chemoradiotherapy (NACRT) could increase the overall number of patients with pCR by 5 (33 vs. 28). Total transformation of tumors by RT to the “hot” state (100/100 BCs) would provide a maximum benefit estimated to 44 pCRs (Model B), thus 16 additional patients in every 100 would reach pCR (44 vs. 28). This increase is solely attributed to the radiovaccination effects of RT. The benefit from the direct effect of RT on cancer cell killing further enhances the number of expected pCRs (in both “hot” and “cold” tumors). An additional benefit from immunotherapy (IO) in patients with immunologically activated tumors is also anticipated, which could have an impact in pCRs and survival of patients.

Given the acquired experimental and clinical knowledge in the field of radiovaccination, preoperative RT could emerge as a crucial tool that would further stimulate immune responses in the already immunologically active TNBC or HER2+ BCs and transform the immunologically “cold” luminal BC into “hot” tumors. In this way, RT-induced lymphocytic infiltration in their tumor microenvironment could be exploited through IO. Moreover, taking into account the enhanced efficacy of NACT in “hot” tumors, regardless of their molecular subtype [17], the induction of dense lymphocytic infiltration to initially “cold” tumors through radiovaccination should improve the interactions between NACT and the immune system; NACT-induced CR should, therefore, be higher (Figure 1).

## 7. Clinical Experience with Preoperative RT

The established practice during the past decades has incorporated RT in the adjuvant setting, while “rescue” RT can be utilized preoperatively in cases of chemotherapy resistant/refractory BC tumors. Early reports of the benefits acquired through the preoperative utilization of radiotherapy for breast cancer date back as early as 1936. Adair F.E. described a significant tumor size reduction (100% of the cases) or even eradication, and a complete clinical response of involved nodes in up to 55% of breast cancer cases when irradiation was performed before surgery [57]. The Stockholm Breast Cancer randomized trial compared the efficacy of preoperative RT followed by mastectomy to mastectomy alone and mastectomy followed by adjuvant RT [58]. It comprised 960 stage I-III BC patients, and both neoadjuvant and adjuvant RT were given in daily fractions of 1.75 Gy for a total of 45 Gy to all regions. Patients submitted to RT had lower locoregional recurrence rates than those treated with surgery alone. The survival rates were significantly better in the preoperative RT group. The combination of chemotherapy with RT has also been examined in a phase III trial by Semiglazov et al. comparing neoadjuvant chemo-RT vs. RT alone for stage IIb-IIIA BC cases. Superior complete regression rates (29.1% vs. 19.4%) and 5-year OS and DFS rates (86.1% vs. 78.3% and 81% vs. 71.6%, respectively) were reported in the neoadjuvant chemo-RT arm [59].

Following the encouraging data from the aforementioned studies, a plethora of retrospective and prospective trials with different chemotherapy regimens and induction chemotherapy followed by chemo-RT or chemo-RT alone were conducted, demonstrating positive results, focusing primarily on the complete response percentages [60]. Moreover, it was shown that initially inoperable locally advanced breast cancer cases were deemed operable after neoadjuvant chemo-RT, while breast-conserving surgery could be a viable option in select cases. Curative surgery for refractory to neoadjuvant chemotherapy-inoperable breast cancer was also feasible after salvage chemo-RT [61]. Two recent retrospective studies evaluated the effects of preoperative RT on the prognosis of BC patients. The Surveillance, Epidemiology, and End Results (SEER) registry analysis of 3708 BC patients selected after propensity score matching adjustment displayed a decreased efficacy of neoadjuvant RT for breast cancer when compared to postoperative irradiation in terms of survival [62]. In 2022, a new SEER analysis confirmed the aforementioned results, especially for stage II and III TNBC patients [63].

## 8. Toward Radiovaccination-Oriented Neoadjuvant Immuno-RT (NA-IRT)

Whether preoperative RT can be used together with IO and NACT in order to improve the survival of BC patients emerges as an appealing hypothesis. Nevertheless, it should be stressed that large RT fields and prolonged fractionated RT applied in BC therapy may mask the effectiveness of a putative radiovaccination effect. A large number of studies confirm that pronounced lymphopenia is a common side effect of RT. This was first reported by Jenkins et al. in 1975, who noticed a reduction in the mean lymphocyte numbers to 31% of the pretreatment values in breast cancer patients undergoing radiotherapy [64]. Several months are required for the restoration of lymphocyte counts after irradiation [65,66]. The effective dose to the circulating immune cells, as estimated by the doses delivered to the lung, heart, and total body, seems to be an important factor defining lymphotoxicity [67]. The magnitude of this lymphopenia has been linked with poor prognosis. In an analysis of 99 patients treated with NACT and postoperative RT who had residual nodal disease after NACT, persistent, low lymphocytic counts one year after RT were linked with regional and distant failure [68]. Hypofractionated schemes of RT seem to be related to a faster recovery of lymphocytes [69,70].

In an attempt to reduce the intensity of lymphopenia during NACT and its combination with RT, large fields of RT and prolonged courses of radiation are, therefore, to be avoided, when possible. The lack of clinical/radiological evidence of node involvement could exclude axillary irradiation in the preoperative setting, while enlarged lymph nodes could be treated with localized fields. Thus, preoperative RT aiming for radiovaccination should be focused on the primary tumor area and/or nodal blocks when present, using partial breast or stereotactic techniques. Indeed, a systematic review by Civil et al. on preoperative partial breast RT for low-risk BC patients concluded that this approach provides high pCR and OS rates and good cosmetic results [71]. Authors also suggested that the omission of surgery emerges as an appealing treatment policy that should be tested in further trials.

Three fractions of 8 Gy is the most common RT schedule applied in experimental and clinical immuno-RT protocols, although different fractionations or even single fractions of ablative or sub-ablative doses can elicit significant immune responses [72]. Consequently, in the context of NA-IRT, RT should be targeted to clinically detectable tumor areas, with a low number of large fractions serving as a potent vaccine. This rationale does not preclude RT from the adjuvant setting. Postoperative breast or chest wall irradiation will be delivered as per the already established guidelines; the difference lies in the fact that a tumor bed boost will have already been prescribed before surgery. However, the necessity of adjuvant RT in cases with pathological complete responses should be further investigated in randomized trials.

**Figure 2:**
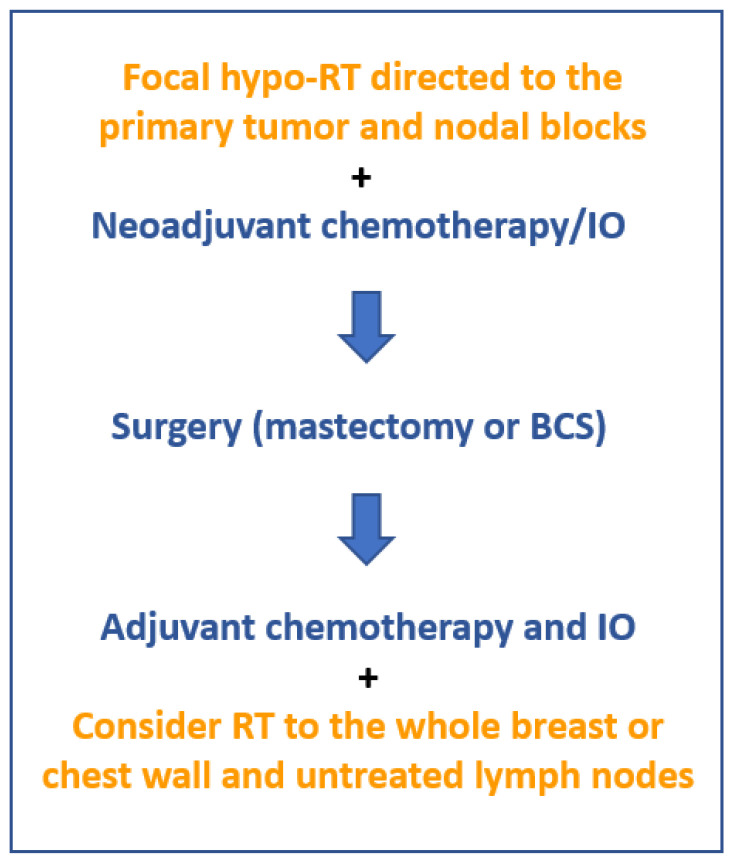
A treatment algorithm incorporating neoadjuvant RT for BC. In order to tap into the radiovaccination properties of irradiation and enhance the efficacy of chemotherapy and IO, neoadjuvant ablative or sub-ablative doses of RT should be targeted to the breast tumor and potential co-existing regional nodal blocks, before or concurrently with chemotherapy and IO. Adjuvant RT could be considered for the whole breast (without a tumor bed boost) or chest wall and previously untreated lymph nodes. (abbreviations: RT = radiotherapy; hypo- = hypofractionated; IO = immunotherapy; BCS = breast-conserving surgery).

The sequencing of neoadjuvant radiotherapy, chemotherapy, and IO should exploit the maximum radiovaccination effect and, of course, the direct effect of RT. Short schedule focal RT, whether directed to the primary tumor and co-existing nodal block areas, could be administered either before or together with the first cycle of chemotherapy and IO. Extensive discussion on this issue has been reported in a recent review article [73]. Figure 2 presents an eventual treatment algorithm that could be tested in randomized trials.

Several ongoing trials have focused on the above rationale. The NCT03366844 phase I/II trial has provided some insight into this topic [74]. Combinations of pembrolizumab and RT (24 Gy in three fractions) followed by NACT for operable TNBC achieved pathological complete responses in three out of five node-positive patients (60%) and was well tolerated [75]. The trial has also expanded to high-risk ER+ and HER2− patients. Focal RT with three 8 Gy fractions given every other day, pembrolizumab and CDX-301 (a dendritic cell and hematopoietic stem cell stimulator) together with neoadjuvant hormonal therapy for stage II-III HER+ and HER2− BC patients, are under investigation in a randomized phase II study (NCT03804944) [76]. Moreover, the P-RAD (NCT04443348) trial studies the efficacy of neoadjuvant RT plus pembrolizumab followed by concurrent chemotherapy and pembrolizumab in HER2- patients [77], while the BreastVAX (NCT04454528) is recruiting patients with early BC that will be submitted to preoperative RT with a single 7 Gy fraction and pembrolizumab IO [78]. A single fraction of 8 Gy together with CMP-001 is also tested in the NCT04807192 trial [79]. CMP-001 is a Toll-like receptor 9 (TLR9) agonist that has been to shown to induce an IFN response and priming of effector T cells [80]. Finally, the Neo-ChekRay (NCT03875573) study aims to assess the safety and treatment outcome with preoperative tumor stereotactic RT (3 × 8 Gy), durvalumab, and oleclumab in luminal B breast carcinomas [81]. Oleclumab is an anti-CD73 antibody that inhibits the production of the immunosuppressive adenosine [82].

## 9. Conclusions

The clinical advances in NACT and the important insights obtained from pathology and biology studies on immune response in BC converge to create new therapeutic concepts highlighting immunological manipulations as essential adjuncts to improving BC prognosis. Drugs that interfere with the tumor microenvironment and the immunosuppressive physicochemical and metabolic conditions emerge as promising policies to improve the efficacy of IO. RT and its high radiovaccination properties could be exploited as a means of enhancing the efficacy of NACT and IO. Focal stereotactic irradiation techniques directed to the primary tumor and involved lymph nodes are a sound option for the RT-NACT-IO combination. A thorough clinical investigation, supported by translation research studies, is necessary to establish such novel treatment approaches in BC.

## Figures and Tables

**Table 1:** Key randomized trials on neoadjuvant systemic therapy for breast cancer patients and major findings.

Author/Year/Reference	No Patients	Randomized Groups	Systemic Therapy	Major Findings
Fisher et al. and Wolmark et al. NSABP B-18 trial (1997 and 2001) [4,5]	1523	Preoperative vs. postoperative chemotherapy	Four cycles of AC	A total of 9.3% and 32% pCR of the primary tumor and lymph nodes, respectively. A total of 9-year OS 70% vs. 69%, and DFS 53% vs. 55%. Higher ipsilateral breast recurrence (10.7% vs. 7.6%) in the preoperative arm (non-significant).
van der Hage et al. EORTC 10902 trial (2001) [6]	698	Preoperative vs. postoperative chemotherapy	FEC chemotherapy	A total of 4.3% pCR. Patients with pCR had a better prognosis. A total of 4-year OS 82% vs. 84%, and DFS 65% vs. 70%. Locoregiοnal recurrence similar in both arms.
Rastogi et al. NSABP-B-18 and B-27 trial (2008) [7]	1560	Preoperative chemotherapy with or without docetaxel	AC and docetaxel followed by surgery vs. AC followed by surgery and postoperative docetaxel	The addition of docetaxel did not improve OS and DFS. Preoperative docetaxel increased the pCR rates (26% vs. 13%). Patients with pCR had a better prognosis.
von Minckwitz et al. GEPARDUO trial (2005) [8]	913	Preoperative chemotherapy with or without docetaxel	Doxorubicin and docetaxel vs. AC followed by docetaxel	pCR rate better in the AC-T arm (14.3% vs. 7.09%). Overall response rates: 78.6% vs. 85%.
Gianni et al. European Cooperative Trial (2009) [9]	1355	Preoperative vs. postoperative chemotherapy	Surgery followed by docetaxel and CMF vs. surgery followed by paclitaxel/doxorubicin and CMF vs. NACT with paclitaxel/doxorubicin and CMF	Adjuvant paclitaxel improved the RFS. No difference between the preoperative and postoperative paclitaxel arms. Patients who achieved pCR in the preoperative chemotherapy arm had a significantly better RFS.
Sikov et al. GALGB 40603 (2015) [11]	443 TNBCs	All patients received NACT. Randomized according to the addition of bevacizumab	NACT with paclitaxel followed by AC with or without paclitaxel and/or Bevacizumab	Addition of carboplatin or bevacizumab significantly increased the pCR (59–60% vs. 44–48%).
von Minckwitz et al. GBG 66 trial (2014) [12]	296 TNBCs and HER2+ carcinomas	All patients received NACT. Randomized according to the administration of carboplatin	NACT with paclitaxel/liposomal doxorubicin with bevacizumab (TNBCs) or Herceptin/lapatinib (HER2+ cases) with or without carboplatin	Carboplatin improved the pCR rates (43.7% vs. 36.9%). In TNBCs carboplatin increased pCRs to 53.2% vs. 36.9%. Carboplatin did not improve pCRs in HER2+ patients.
Schmid et al. KEYNOTE-522 trial (2020, 2022) [19,20]	1174 TNBCs	All patients received neoadjuvant systemic therapy. Randomized according to the administration of pembrolizumab	NACT with AC with or without pembrolizumab	Increased pCRs in PD-L1 positive tumors (68.9% vs. 54.9%). Increased pCRs in PD-L1 negative tumors (45.3% vs. 30.3%). Reduction in progression at any cite by pembrolizumab (7.4% vs. 11.8%). Estimated EFS at 36 months was 84.5% vs. 76.8% in favor of pembrolizumab.
Nanda et al. I-SPY2 trial (2020) [21]	250 MammaPrint-high-risk luminal/HER2− BC and TNBC	All patients received neoadjuvant systemic therapy. Randomized according to the administration of pembrolizumab	Taxane- and anthracycline-based NACT with or without pembrolizumab	Increased pCRs in TNBCs (60% vs. 22%). Increased pCRs in luminal/HER2− cases (30% vs. 13%). Unknown effect on survival.
Mittendorf et al. IMpassion 031 trial (2020) [22]	333 TNBCs	All patients received neoadjuvant systemic therapy. Randomized according to the administration of atezolizumab	NACT with Nab-paclitaxel followed by AC with or without atezolizumab	Improved pCRs (57.6% vs. 41.1%). Unknown effect on survival.

Abbreviations: NACT = neoadjuvant chemotherapy; AC = doxorubicin–cyclophosphamide; TNBC = triple-negative breast cancer; pCR = pathological complete response; OS = overall survival; DFS = disease-free survival; FEC = fluorouracil–epirubicin–cyclophosphamide; CMF = cyclophosphamide–methotrexate–fluorouracil; RFS = relapse-free survival; EFS = event-free survival.

## Data Availability

Not applicable.
